# Impact of the diagnosis-to-treatment interval on the survival of patients with CD5-positive diffuse large B-cell lymphoma

**DOI:** 10.1007/s00277-026-07021-0

**Published:** 2026-04-25

**Authors:** Yuma Nato, Kana Miyazaki, Dai Maruyama, Hiroyuki Takahashi, Kazutaka Sunami, Eiju Negoro, Satsuki Murakami, Takahiro Okada, Nobuyuki Takayama, Yuri Miyazawa, Ilseung Choi, Shuji Momose, Yuto Kaneda, Masahiro Yoshida, Naoto Tomita, Tohru Murayama, Momoko Nishikori, Junji Hiraga, Kohtaro Toyama, Naoki Takahashi, Taro Masunari, Jun Takizawa, Isao Tawara, Naoko Asano, Koichi Ohshima, Koji Izutsu, Koji Kato, Ritsuro Suzuki, Motoko Yamaguchi

**Affiliations:** 1 Department of Hematological Malignancies, Mie University Graduate School of Medicine, Tsu, Japan; 2 Department of Hematology and Oncology, Mie University Graduate School of Medicine, 2-174 Edobashi, Tsu, Mie 514-8507 Japan; 3 Department of Hematology Oncology, Cancer Institute Hospital, Japanese Foundation for Cancer Research, Tokyo, Japan; 4 Department of Hematology and Medical Oncology, Kanagawa Cancer Center, Yokohama, Japan; 5 Department of Hematology, NHO Okayama Medical Center, Okayama, Japan; 6 Department of Hematology and Oncology, University of Fukui, Fukui, Japan; 7 Department of Hematology, Aichi Medical University, Nagakute, Japan; 8 Department of Hematology and Oncology, Shimane University School of Medicine, Izumo, Japan; 9 Department of Hematology, Kyorin University School of Medicine, Mitaka, Japan; 10 Department of Hematology, Gunma University Graduate School of Medicine, Maebashi, Japan; 11 Department of Hematology and Cell Therapy, NHO Kyushu Cancer Center, Fukuoka, Japan; 12 Department of Pathology, Saitama Medical Center, Saitama Medical University, Kawagoe, Japan; 13 Department of Hematology and Infectious Disease, Gifu University Hospital, Gifu, Japan; 14 Department of Hematology, Osaka City General Hospital, Osaka, Japan; 15 Department of Hematology and Oncology, St. Marianna University School of Medicine, Kawasaki, Japan; 16 Department of Hematology, Hyogo Cancer Center, Akashi, Japan; 17 Department of Hematology, Graduate School of Medicine, Kyoto University, Kyoto, Japan; 18 Department of Hematology, Toyota Kosei Hospital, Toyota, Japan; 19 Department of Hematology, Fujioka General Hospital, Gunma, Japan; 20 Department of Hematology, International Medical Center, Saitama Medical University, Hidaka, Japan; 21 Department of Hematology/Infectious Diseases, Chugoku Central Hospital, Fukuyama, Japan; 22 Department of Hematology, Endocrinology and Metabolism, Faculty of Medicine, Niigata University, Niigata, Japan; 23 Department of Molecular Diagnostics, Nagano Prefectural Shinshu Medical Center, Suzaka, Japan; 24 Department of Pathology, Kurume University School of Medicine, Kurume, Japan; 25 Department of Hematology, National Cancer Center Hospital, Tokyo, Japan; 26 Department of Medicine and Biosystemic Science, Kyushu University Graduate School of Medical Sciences, Fukuoka, Japan

**Keywords:** CD5 antigen, Diffuse large B-cell lymphoma, Diagnosis-to-treatment interval, DA-EPOCH-R

## Abstract

We analyzed the impact of the diagnosis-to-treatment interval (DTI) on survival in patients with CD5-positive diffuse large B-cell lymphoma (CD5 + DLBCL), using a data set of newly diagnosed patients. Among the 336 eligible patients, 247 (74%) received R-CHOP (rituximab, cyclophosphamide, doxorubicin, vincristine, and prednisolone), and 89 (26%) were treated with dose-adjusted (DA)-EPOCH-R (etoposide, prednisolone, vincristine, cyclophosphamide, doxorubicin, and rituximab). The median DTI was 18 days (range 0–118). The short DTI (≤ 14 days) group included 135 patients (40%), and the long DTI (> 14 days) group included 201 patients (60%). Compared with the long DTI group, the short DTI group had more aggressive disease characteristics. Both the progression-free survival (PFS) (*P* = 0.01) and the overall survival (OS) (*P* < 0.01) were significantly inferior in the short DTI group compared with those in the long DTI group. Among 89 patients who received DA-EPOCH-R, no significant differences in PFS (*P* = 0.92) or OS (*P* = 0.86) were observed between the two groups. Multivariate analysis revealed that no DA-EPOCH-R was a risk factor for PFS in the short DTI group (*P* = 0.06). A short DTI was a negative prognostic factor in our CD5 + DLBCL cohort. DA-EPOCH-R could be considered a potential treatment option for patients with a short DTI.

**Supplementary Information:**

The online version contains supplementary material available at 10.1007/s00277-026-07021-0.

## Introduction

CD5-positive diffuse large B-cell lymphoma (CD5 + DLBCL) comprises 5–10% of DLBCL-not otherwise specified (NOS) cases and is characterized by aggressive clinical features [1–5]. Up to 80% of CD5 + DLBCL cases are classified as the activated B-cell (ABC) type [5–7], and myeloid differentiation primary response 88 (*MYD88*) (L265P) and *CD79B* mutations are detected in 33–52% and 38% of CD5 + DLBCL cases, respectively [8, 9]. Patients with CD5 + DLBCL experience significantly shorter survival than those with CD5-negative DLBCL after treatment with rituximab, cyclophosphamide, doxorubicin, vincristine, and prednisolone (R-CHOP) [10–15]; moreover, they have a high incidence of central nervous system (CNS) relapse (13%) [4]. We conducted a phase II study of dose-adjusted (DA)-EPOCH-R (etoposide, prednisolone, vincristine, cyclophosphamide, doxorubicin, and rituximab) and high-dose methotrexate (HD-MTX) (DA-EPOCH-R/HD-MTX) for patients newly diagnosed with stage II–IV CD5 + DLBCL (UMIN000008507) to address this poor prognosis [16, 17]. Although DA-EPOCH-R/HD-MTX was selected for more patients ≤ 60 years old and for those whose Eastern Cooperative Oncology Group (ECOG) performance status (PS) was > 1, the favorable survival and manageable toxicity of DA-EPOCH-R/HD-MTX were subsequently validated in a large multicenter retrospective cohort [18]. Due to the complexity and the requirement for hospitalization, there is heterogeneity in treatment options for patients with untreated CD5 + DLBCL in clinical settings.

The diagnosis-to-treatment interval (DTI) is defined as the time between the diagnosis and the initiation of treatment. A shorter DTI is a risk factor for aggressive non-Hodgkin lymphomas [19, 20]. In patients with newly diagnosed DLBCL, the DTI is strongly associated with prognostic clinical factors and outcomes [21]. A shorter DTI is related to more aggressive disease characteristics, a larger tumor volume, and poorer outcomes in patients with DLBCL [21–24]. Although a short DTI is a risk factor in unselected DLBCL, the impact of the DTI may differ in patients with CD5 + DLBCL. The effect of treatment options, such as DA-EPOCH-R, remains unknown. We analyzed the data collected from a multicenter retrospective study of patients diagnosed with CD5 + DLBCL to determine the association between the DTI and survival.

## Patients and methods

### Patients

CD5 + DLBCL new-era (UMIN-CTR: UMIN000049460) [18] was a multicenter retrospective study of 413 patients who were consecutively diagnosed with untreated CD5 + DLBCL from 2016 to 2021 at 30 hospitals in Japan. This study was conducted in accordance with the Declaration of Helsinki and was approved by the institutional review board of Mie University Graduate School of Medicine (approval number: H2022-204). The requirement for informed consent was waived because of the retrospective nature of the study. Patient selection and detailed cytogenetic evaluation are summarized in the Supplementary Methods.

### Analysis of the DTI

The DTI was defined as the number of days from the date of diagnosis to the initiation of immunochemotherapy. The date of diagnosis was defined as the date when the first biopsy specimen containing lymphoma was obtained. In accordance with a previous report [21], we considered patients whose DTI was 0 to 14 days as the short DTI group and those whose DTI was more than 14 days as the long DTI group. The rationale for the cutoff of 14 days for the DTI and the statistical analyses are included in the Supplemental Methods.

## Results

### Patient characteristics and distribution of the DTI

**Fig. 1:**
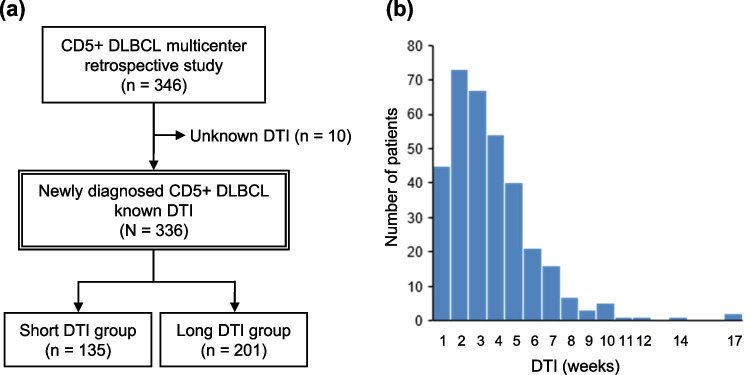
Patient selection (**a**) and distributions of the DTI (**b**). CD5 + DLBCL, CD5-positive diffuse large B-cell lymphoma; DTI, diagnosis-to-treatment interval

Among the 346 patients, 10 were excluded because of a lack of DTI data. Overall, 336 patients were eligible (Fig. 1a). The baseline characteristics of all 336 patients are listed in Table 1. The median age at diagnosis was 71 years (range, 23–92). The median DTI was 18 days (range: 0–118). The histogram of the DTI is shown in Fig. 1b. The median follow-up time was 42 months. Kaplan‒Meier PFS and OS curves grouped by weekly DTI are shown in Figure SIa, b. Among the 99 patients whose maximum tumor diameter was 5 cm or larger, a significant inverse correlation was observed between the DTI and the maximum tumor diameter (Spearman’s correlation rho = −0.26; *P* = 0.009) (Figure SII). First-line treatment, distribution of the DTI, and survival stratified by weekly DTI are summarized in the Supplementary Results.

**Table 1:** Patient demographics and baseline clinical characteristics of the entire cohort (*n* = 336)

Characteristic	All patients (*n* = 336) n (%)	Short DTI (*n* = 135) n (%)	Long DTI (*n* = 201) n (%)	P*
Age (years)				
Median (range)	71 (23—92)	71 (23—89)	70 (29—92)	
> 60 years	257 (76)	105 (78)	152 (76)	0.70
Sex				
Male	181 (54)	81 (60)	100 (50)	0.07
Stage				
III-IV	248 (74)	123 (91)	125 (62)	< 0.01
ECOG PS				
> 1	103 (31)	66 (49)	37 (18)	< 0.01
Extranodal site(s)				
> 1	141 (42)	74 (55)	67 (33)	< 0.01
Serum LDH				
Elevated	240 (71)	117 (87)	123 (61)	< 0.01
B symptoms				
Present	97 (29)	66 (49)	31 (16)	< 0.0
Unknown	4	1	3	1
Size				
≥ 10 cm	27 (8)	16 (12)	11 (5)	0.041
COO (Hans)				
GCB	91 (34)	30 (28)	61 (38)	0.09
Non-GCB	178 (66)	78 (72)	100 (62)	
Unknown	67	27	40	
IPI				
Low	58 (17)	11 (8)	47 (23)	
Low-int	63 (19)	12 (9)	51 (25)	
High-int	87 (26)	31 (23)	56 (28)	
High	128 (38)	81 (60)	47 (23)	

*DTI* diagnosis-to-treatment interval, *ECOG PS* Eastern Cooperative Group Performance Status, *LDH* lactate dehydrogenase, *COO* cell-of-origin, *GCB* germinal center B, *IPI* International Prognostic Index, *int* intermediate

*short DTI vs. long DTI

### *Survival in the entire CD5* + *DLBCL cohort: short DTI vs. long DTI*

In total, 135 patients were classified into the short DTI group, and 201 patients were classified into the long DTI group (Table 1). More patients with stage III/IV disease, an ECOG PS > 1, > 1 extranodal sites, elevated serum lactate dehydrogenase (LDH) levels, B symptoms, and a maximum tumor diameter ≥ 10 cm were identified in the short DTI group than in the long DTI group.

**Fig. 2:**
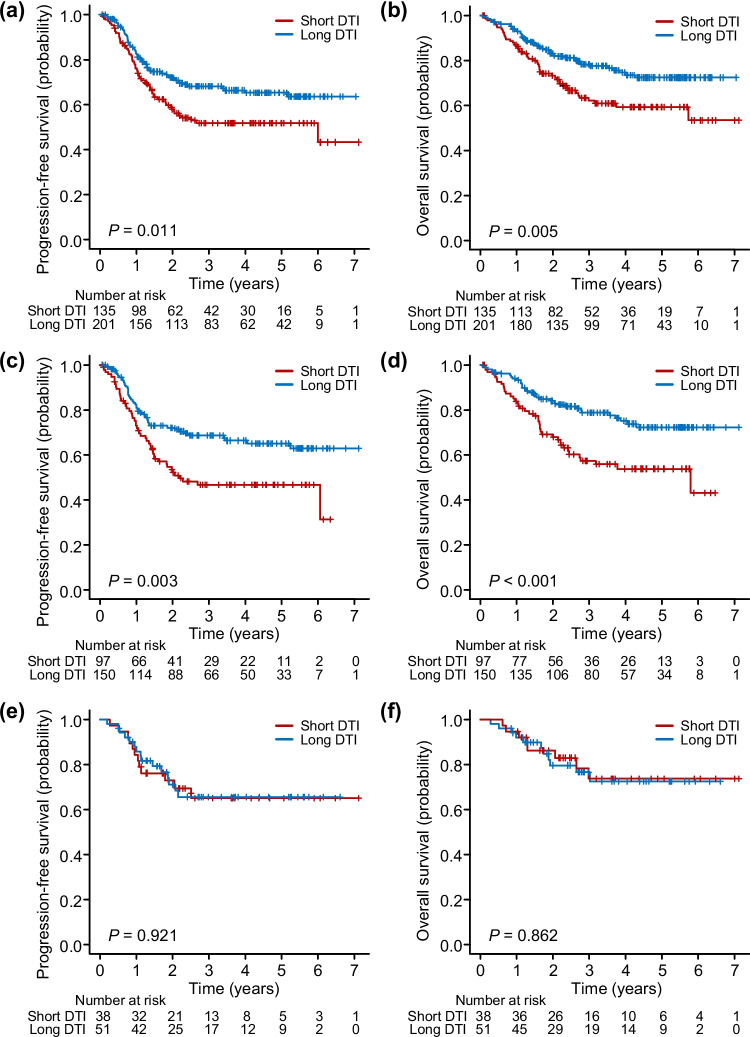
Kaplan‒Meier curves of the progression-free survival and overall survival of the short DTI group vs. the long DTI group in the entire cohort (**a**, **b**), in the patients who received R-CHOP (**c**, **d**), and in the patients who received DA-EPOCH-R (**e**, **f**). DTI, diagnosis-to-treatment interval; R-CHOP, rituximab, cyclophosphamide, doxorubicin, vincristine, and prednisolone; DA-EPOCH-R, dose-adjusted etoposide, prednisolone, vincristine, cyclophosphamide, doxorubicin, and rituximab

In the short and long DTI groups, the 2-year PFS rates were 58% and 72%, respectively, and the 2-year OS rates were 73% and 83%, respectively. Both the PFS (*P* = 0.01) and OS (*P* = 0.005) of the short DTI group were significantly shorter than those of the long DTI group (Fig. 2a, b).

### Survival of the patients in the R-CHOP and DA-EPOCH-R cohorts: short DTI vs. long DTI

Among the 336 eligible patients, 247 (74%) received R-CHOP, and 89 (26%) were treated with DA-EPOCH-R. Among the 247 patients who received R-CHOP, 97 were classified in the short DTI group, and 150 were classified in the long DTI group, as shown in Table 2. More patients with stage III/IV disease, ECOG PS > 1, > 1 extranodal sites, elevated serum LDH levels, and B symptoms were identified in the short DTI group than in the long DTI group. The 2-year PFS rates were 52% and 72%, and the 2-year OS rates were 68% and 84%, in the short and long DTI groups, respectively. Both the PFS (*P* = 0.003) and OS (*P* < 0.001) of the short DTI group were significantly shorter than those of the long DTI group (Fig. 2c, d).

**Table 2:** Patient demographics and baseline clinical characteristics by treatment

Characteristic	R-CHOP (*n* = 247)			DA-EPOCH-R (*n* = 89)		
	Short DTI (*n* = 97) n (%)	Long DTI (*n* = 150) n (%)	P*	Short DTI (*n* = 38) n (%)	Long DTI (*n* = 51) n (%)	P**
Age (years)						
Median (range)	73 (34—89)	71 (39—92)		65 (23—75)	65 (29—80)	
> 60 years	83 (86)	117 (78)	0.18	22 (58)	35 (69)	0.37
Sex						
Male	61 (63)	75 (50)	0.051	20 (53)	25 (49)	0.83
Stage						
III-IV	88 (91)	92 (61)	< 0.01	35 (92)	33 (65)	< 0.01
ECOG PS						
> 1	48 (50)	21 (14)	< 0.01	18 (47)	16 (31)	0.19
Extranodal site(s)						
> 1	49 (51)	46 (31)	< 0.01	25 (66)	21 (41)	0.03
Serum LDH						
Elevated	83 (86)	84 (56)	< 0.01	34 (90)	39 (77)	0.16
B symptoms						
Present	43 (44)	21 (14)	< 0.01	23 (62)	10 (20)	< 0.01
Unknown	0	2		1	1	
Size						
≥ 10 cm	11 (12)	7 (5)	0.08	5 (13)	4 (8)	0.49
COO (Hans)						
GCB	25 (32)	48 (40)	0.29	5 (17)	13 (33)	0.18
Non-GCB	54 (68)	73 (60)		24 (83)	27 (68)	
Unknown	18	29		9	11	
IPI						
Low	8 (8)	36 (24)		3 (8)	11 (22)	
Low-int	8 (8)	39 (26)		4 (11)	12 (24)	
High-int	20 (21)	48 (32)		11 (29)	8 (16)	
High	61 (63)	27 (18)		20 (53)	20 (39)	

*R-CHOP* rituximab, cyclophosphamide, doxorubicin, vincristine, and prednisolone, *DA-EPOCH-R* dose-adjusted etoposide, prednisolone, vincristine, cyclophosphamide, doxorubicin and rituximab, *DTI* diagnosis-to-treatment interval, *ECOG PS* Eastern Cooperative Group Performance Status, *LDH* lactate dehydrogenase, *COO* cell-of-origin, *GCB* germinal center B, *IPI* International Prognostic Index, *int* intermediate

*short DTI vs. long DTI in patients who received R-CHOP, **short DTI vs. long DTI in patients who were treated with DA-EPOCH-R

Among the 89 patients who received DA-EPOCH-R, 38 were included in the short DTI group, and 51 were included in the long DTI group, as shown in Table 2. More patients with stage III/IV disease, > 1 extranodal sites, and B symptoms were identified in the short DTI group than in the long DTI group. The 2-year PFS rates were 73% and 71%, and the 2-year OS rates were 86% and 80%, in the short and long DTI groups, respectively. No significant differences in either PFS (*P* = 0.92) or OS (*P* = 0.86) were observed between the two groups (Fig. 2e, f). The survival of the patients who were treated with R-CHOP vs. DA-EPOCH-R in the short and long DTI groups is summarized in the Supplementary Results.

### Adverse events of the patients in the short DTI group: R-CHOP vs. DA-EPOCH-R

The incidences of Grade 3 to 4 adverse events in the short DTI group are listed in Table 3. Among the 97 patients who received R-CHOP, the most common nonhematologic toxicity was infection (13%). Among the 38 patients who were treated with DA-EPOCH-R, the most common nonhematologic toxicity was infection (16%). Febrile neutropenia was observed in 27% and 42% of patients who received R-CHOP and DA-EPOCH-R, respectively. The adverse events experienced by the patients in the long DTI group are summarized in the Supplemental Results.

**Table 3:** Grade 3/4 adverse events of the patients in the short DTI group by treatment regimen

Adverse events	R-CHOP (*n* = 97)		DA-EPOCH-R (*n* = 38)	
	Grade 3 n (%)	Grade 4 n (%)	Grade 3 n (%)	Grade 4 n (%)
Hematologic				
Neutropenia	13 (13)	62 (64)	2 (5)	28 (74)
Leukopenia	24 (25)	45 (46)	4 (11)	25 (66)
Thrombocytopenia	16 (16)	17 (18)	12 (32)	9 (24)
Anemia	27 (28)	5 (5)	21 (55)	3 (79)
Febrile neutropenia	23 (24)	3 (3)	16 (42)	0
Nonhematologic				
AST increased	0	1 (1)	3 (8)	0
ALT increased	1 (1)	1 (1)	3 (8)	0
Hyponatremia	6 (6)	0	2 (5)	0
Constipation	5 (5)	0	1 (3)	0
Nausea	4 (4)	0	1 (3)	0
Infection	10 (10)	3 (3)	4 (11)	2 (5)
Allergic reaction	0	0	1 (3)	0
Tumor lysis syndrome	1 (1)	0	1 (3)	1 (3)
Sensory neuropathy	0	0	0	0
Motor neuropathy	0	0	0	0
Pneumonitis	1 (1)	0	1 (3)	0
Thrombosis	3 (3)	0	0	0
Others	3 (3)	1 (1)	3 (8)	0

*DTI* diagnosis-to-treatment interval, *R-CHOP* rituximab, cyclophosphamide, doxorubicin, vincristine, and prednisolone, *DA-EPOCH-R* dose-adjusted etoposide, prednisolone, vincristine, cyclophosphamide, doxorubicin and rituximab, *AST* aspartate aminotransferase, *ALT* alanine aminotransferase

Others: In patients treated with the R-CHOP regimen, Grade 4 gastrointestinal hemorrhage (*n* = 1), Grade 3 vasculitis (*n* = 1), Grade 3 urinary retention (*n* = 1), and Grade 3 urinary tract infection (*n* = 1) were observed. In patients treated with the DA-EPOCH-R regimen, Grade 3 gastrointestinal disorders—other, specific (intestinal emphysema) (*n* = 1), Grade 3 oral pain (*n* = 1), and Grade 3 fatigue (*n* = 1) were observed

### Univariate and multivariate analyses of the short DTI group

A univariate analysis of the PFS of the 135 patients in the short DTI group revealed that an elevated serum LDH level was a risk factor, and age > 60 years, ECOG PS > 1, stage III/IV, and no DA-EPOCH-R tended to indicate relapse. A multivariate analysis of PFS in the short-DTI group revealed that DA-EPOCH-R tended to be a prognostic factor (HR, 0.53; 95% CI, 0.27–1.03;* P* = 0.06) (Table 4).

**Table 4:** Univariate and multivariate analyses for factors affecting the PFS or OS of the short DTI group (*n* = 135)

Variable for PFS	Univariate			Multivariate		
	HR	95% CI	*P*	HR	95% CI	*P*
Age > 60 years	1.88	0.95—3.71	0.07	1.30	0.63—2.69	0.48
Elevated serum LDH	3.84	1.20—12.3	0.02	2.80	0.81—9.73	0.11
ECOG PS > 1	1.56	0.94—2.60	0.09	1.36	0.81—2.29	0.25
Stage III or IV	3.54	0.86—14.5	0.08	2.16	0.49—9.53	0.31
Extranodal sites > 1	1.11	0.67–1.85	0.69	―	―	―
DA-EPOCH-R	0.53	0.28–1.00	0.051	0.53	0.27—1.03	0.06

Variable for OS	Univariate			Multivariate		
	HR	95% CI	*P*	HR	95% CI	*P*
Age > 60 years	4.27	1.53—11.9	0.006	3.00	1.04—8.69	0.04
Elevated serum LDH	2.99	0.93—9.66	0.06	2.19	0.66—7.29	0.20
ECOG PS > 1	1.68	0.95—2.98	0.07	1.55	0.86—2.79	0.14
Stage III or IV	2.58	0.63—10.6	0.19	―	―	―
Extranodal sites > 1	1.63	0.91—2.93	0.10	―	―	―
DA-EPOCH-R	0.44	0.21—0.94	0.03	0.51	0.23 −1.12	0.09

*PFS* progression-free survival, *OS* overall survival, *DTI* diagnosis-to-treatment interval, *HR* hazard ratio, *CI* confidence interval, *LDH* lactate dehydrogenase, *ECOG PS* Eastern Cooperative Group Performance Status, *DA-EPOCH-R* dose-adjusted etoposide, prednisolone, vincristine, cyclophosphamide, doxorubicin, and rituximab

A univariate analysis of the OS of the 135 patients in the short DTI group revealed that age > 60 years and no treatment with DA-EPOCH-R were risk factors, and elevated serum LDH levels and an ECOG PS > 1 tended to be risk factors. A multivariate analysis of OS revealed that age > 60 years (HR, 3.00; 95% CI, 1.04–8.69; *P* = 0.04) was an independent prognostic factor, and DA-EPOCH-R treatment tended to be a prognostic factor (HR, 0.51; 95% CI, 0.23–1.12; *P* = 0.09) (Table 4).

### Survival of the patients who were older than 60 years in the DA-EPOCH-R cohort

Among the 89 patients who received DA-EPOCH-R, 57 were older than 60 years, and 32 were ≤ 60 years old. The 2-year PFS rates were 66% and 74%, and the 2-year OS rates were 75% and 92%, respectively. The OS of the patients who were older than 60 years was significantly shorter than that of the patients who were ≤ 60 years old (*P* = 0.01). No significant difference in PFS was observed between the two groups (*P* = 0.34).

## Discussion

To our knowledge, this study is the first to evaluate the impact of the DTI on the survival of patients with CD5 + DLBCL. In our cohort, patients in the short DTI group presented with more aggressive features requiring urgent treatment. As previously reported in patients with DLBCL [21], a short DTI was confirmed to be a negative prognostic factor in patients with CD5 + DLBCL. DA-EPOCH-R may have mitigated the adverse prognostic impact of a short DTI on our CD5 + DLBCL cohort.

Our findings suggest that DA-EPOCH-R treatment may overcome the adverse prognostic implications of the DTI. This conclusion is based on the finding of no significant differences in PFS or OS between the short and long DTI groups among the 89 patients who were treated with DA-EPOCH-R. Similar results were reported in a retrospective study of DA-EPOCH-R for patients with untreated large B-cell lymphoma from a single institution (*n* = 190, including 22 patients with primary mediastinal large B-cell lymphoma) [25]. For the early diagnosis of lymphoma, rapid detection using flow cytometry can identify the immunophenotype [26], although a diagnosis based on light-chain restriction from flow cytometry alone is controversial [27, 28]. In our cohort, patients with a DTI within 7 days might have been diagnosed using flow cytometry.

A short DTI is associated with aggressive molecular phenotypes of DLBCL, such as a double-hit signature [29]. Since the efficacy of DA-EPOCH-R against double-hit lymphoma was validated in a phase II study and a subgroup analysis of CALGB 50303 suggested that the PFS of patients with an IPI > 2 in the DA-EPOCH-R arm improved, DA-EPOCH-R was considered effective for patients with high-risk features [30, 31]. Despite the benefit of DA-EPOCH-R, the prognosis of patients with a short DTI is expected to improve further if more effective treatments are developed. Recent efforts in DLBCL treatment have focused on genetic mutation-specific strategies, as shown in the GUIDANCE-01 trial [32]. The addition of a Bruton’s tyrosine kinase inhibitor is expected to improve the prognosis for patients with CD5 + DLBCL [33]. The prognostic impact of the DTI on CD5 + DLBCL may also change in the future because of these targeted treatment approaches. Accumulating evidence suggests that bispecific antibodies show promising efficacy in the treatment of DLBCL [34, 35]. The negative impact of a short DTI may be overcome by front-line therapy, including bispecific antibodies.

Our study has several limitations. First, the results were based on a retrospective analysis. Second, our study did not include patients who received polatuzumab vedotin plus R-CHP [36]. Another limitation is that some cases of double/triple-hit lymphoma remained undetected. Nevertheless, in conclusion, our present study is the first to reveal the effect of the DTI on the survival of patients with CD5 + DLBCL. Importantly, our findings suggest that DA-EPOCH-R may mitigate the adverse prognostic effect of a short DTI in our cohort. Further investigations are warranted to clarify this relationship.

## Supplementary Information

Below is the link to the electronic supplementary material.

Supplementary file1 (DOCX 70 kb)

Supplementary file2 (PPTX 165 kb) Figure SI. Kaplan‒Meier curves of the progression-free survival and overall survival of patients stratified by DTI grouped by week for the entire cohort (a, b), for the patients who received R-CHOP (c, d), and for the patients who received DA-EPOCH-R (e, f). DTI, diagnosis-to-treatment interval; R-CHOP, rituximab, cyclophosphamide, doxorubicin, vincristine, and prednisolone; DA-EPOCH-R, dose-adjusted etoposide, prednisolone, vincristine, cyclophosphamide, doxorubicin, and rituximab

Supplementary file3 (PPTX 27 kb) Figure SII. Correlations between the DTI and the maximum tumor diameter were determined using Spearman’s rank correlation analysis. DTI, diagnosis-to-treatment interval

Supplementary file4 (PPTX 106 kb) Figure SIII. Distributions of the DTI of patients who received R-CHOP (a) and DA-EPOCH-R (b). Kaplan‒Meier curves of the progression-free survival and overall survival of patients who received R-CHOP compared with those who received DA-EPOCH-R in the short DTI group (c, d) and in the long DTI group (e, f). DTI, diagnosis-to-treatment interval; R-CHOP, rituximab, cyclophosphamide, doxorubicin, vincristine, and prednisolone; DA-EPOCH-R, dose-adjusted etoposide, prednisolone, vincristine, cyclophosphamide, doxorubicin, and rituximab

## Data Availability

The data sets analyzed during the current study are available from the corresponding author on reasonable request.
