# Supplementary material for: Impact of the diagnosis-to-treatment interval on the survival of patients with CD5-positive diffuse large B-cell lymphoma
Source: Ann Hematol. 2026 Apr 25;105(5):267. doi: 10.1007/s00277-026-07021-0 (PMC13110205; doi:10.1007/s00277-026-07021-0)
Supplement: Supplementary file 1 — Supplementary file1 (DOCX 70 kb) [file 277_2026_7021_MOESM1_ESM.docx]

**Supplemental Material**

**Impact of the diagnosis-to-treatment interval on the survival of patients with CD5-positive diffuse large B-cell lymphoma**

Yuma Nato,^1,2^ Kana Miyazaki,^2^ Dai Maruyama,^3^ Hiroyuki Takahashi,^4^ Kazutaka Sunami,^5^ Eiju Negoro,^6^ Satsuki Murakami,^7^ Takahiro Okada,^8^ Nobuyuki Takayama,^9^ Yuri Miyazawa,^10^ Ilseung Choi,^11^ Shuji Momose,^12^ Yuto Kaneda,^13^ Masahiro Yoshida,^14^ Naoto Tomita,^15^ Tohru Murayama,^16^ Momoko Nishikori,^17^ Junji Hiraga,^18^ Kohtaro Toyama,^19^ Naoki Takahashi,^20^ Taro Masunari,^21^ Jun Takizawa,^22^ Isao Tawara,^2^ Naoko Asano,^23^ Koichi Ohshima,^24^ Koji Izutsu,^25^ Koji Kato,^26^ Ritsuro Suzuki,^8^ and Motoko Yamaguchi^1^

*Authors' affiliations:* as in the article

**Contact information for correspondence:**

Kana Miyazaki

E-mail: kmiyazaki@clin.medic.mie-u.ac.jp

**The following sections are included:**

Supplementary Methods Pages 2 to 3

Supplementary Results Pages 4 to 5

Supplementary Table SI to SII Pages 6 to 7

**Supplemental Methods**

**Patient selection and detailed cytogenetic evaluation**

Among the 413 patients, 22 were excluded because of an ineligible diagnosis or insufficient clinical information, 18 had CNS involvement at diagnosis, and 27 did not receive any anthracycline-containing chemotherapy with rituximab. Overall, 346 patients were deemed eligible for the previous study. In the present study, patients whose DTI was unknown were excluded.

MYC rearrangements were evaluated by fluorescence in situ hybridization (FISH) (n = 35) and G-banding (n = 159). G-banding results were obtained for 77 cases, and no results were obtained for 82 cases because of poor proliferation. MYC rearrangement was documented in 5 cases: two by FISH and three by G-banding. No patient presented with double/triple-hit lymphoma according to the institutional diagnosis. No patient had a past medical history of lymphoproliferative disorders, including chronic lymphocytic leukemia or small lymphocytic lymphoma.

**Rationale for the cutoff of 14 days for the diagnosis-to-treatment interval**

The cutoff of 14 days for the diagnosis-to-treatment interval (DTI) was determined on the basis of the median DTI of the discovery cohort in the University of Iowa and Mayo Clinic Specialized Programs of Research Excellence Molecular Epidemiology Resource (MER). In accordance with the statistical protocol, clinical variables were analyzed by dividing the discovery data set into two groups at this median point. Patients whose DTI was 0 to 14 days were significantly associated with aggressive clinical features, including elevated serum lactate dehydrogenase levels, an Eastern Cooperative Oncology Group performance status > 1, the presence of B symptoms, and bulky disease. Although the median DTI of 23 days in the Lymphoma Study Association LNH-2003 clinical trial program was longer than that in MER, the cutoff of 14 days consistently identified a subgroup with poor prognosis [1].

**Statistical analysis**

OS was defined as the time from the date of diagnosis to the date of death from any cause. PFS was defined as the time from the date of diagnosis to the earliest date of progression, relapse, or death from any cause. Toxicity was graded according to the Common Terminology Criteria for Adverse Events version 5.0. The correlation between the DTI and the maximum tumor diameter was evaluated using Spearman’s rank correlation analysis. The distributions of variables between the two groups were assessed using Fisher’s exact test. Survival estimates were calculated using the Kaplan‒Meier method. Risk factors for OS and PFS were identified using the Cox proportional hazards method. All P values were two-sided and had an overall significance level of 0.05. Statistical analyses were performed using the SPSS version 29 (IBM, NY, USA) package and EZR version 1.64 [2].

**References**

1. Maurer MJ, Ghesquières H, Link BK et al. (2018) Diagnosis-to-Treatment Interval Is an Important Clinical Factor in Newly Diagnosed Diffuse Large B-Cell Lymphoma and Has Implication for Bias in Clinical Trials. J Clin Oncol 36:1603-1610.

2. Kanda Y (2013) Investigation of the freely available easy-to-use software 'EZR' for medical statistics. Bone Marrow Transplant 48:452-458.

**Supplemental Results**

**First-line treatment, distribution of DTI, and survival stratified by weekly DTI**

Among the 336 eligible patients, 247 received R-CHOP, including R-pirarubicin-COP, and 89 were treated with DA-EPOCH-R as a first-line treatment (Table 2). The median DTI was 19 days (range: 0–112) and 17 days (range: 0–118) for the patients who received R-CHOP and DA-EPOCH-R, respectively. Histograms of the DTI results stratified by first-line treatment are shown in Figure SII. The Kaplan‒Meier PFS and OS curves of the patients who were treated with R-CHOP and DA-EPOCH-R grouped by each week of DTI are shown in Figure SIII a, b.

**Survival of the patients in the short and long DTI groups: R-CHOP vs. DA-EPOCH-R**

Among the 135 patients in the short DTI group, 97 received R-CHOP, and 38 received DA-EPOCH-R (Table SI). More patients were > 60 years old in the R-CHOP cohort than in the DA-EPOCH-R cohort. In the patients who received R-CHOP and DA-EPOCH-R, the 2-year PFS rates were 52% and 73% and the 2-year OS rates were 68% and 86%, respectively. Both the PFS (*P* = 0.047) and OS (*P* = 0.030) of the patients who received R-CHOP were significantly shorter than that of those who received DA-EPOCH-R (Figure SIII c, d).

Among the 201 patients in the long DTI group, 150 patients were treated with R-CHOP, and 51 patients were treated with DA-EPOCH-R (Table SI). More patients with an ECOG PS > 1 and elevated serum LDH levels were identified in the DA-EPOCH-R cohort than in the R-CHOP cohort. The 2-year PFS rates were 72% and 71% and the 2-year OS rates were 84% and 80% in the patients who were treated with R-CHOP and DA-EPOCH-R, respectively. No significant differences in either PFS (*P* = 0.80) or OS (*P* = 0.90) were observed between the two groups (Figure III e, f).

**Adverse events of the patients in the long DTI group: R-CHOP vs. DA-EPOCH-R**

In the long DTI group, the incidences of Grade 3 to 5 adverse events are listed in Supplemental Table 2. Among the 150 patients who received R-CHOP, the Grade 5 nonhematologic toxicity was pneumonitis (1%), and the most common Grade 3 to 4 nonhematologic toxicity was infection (5%). Among the 51 patients who were treated with DA-EPOCH-R, the most common Grade 3 to 4 nonhematologic adverse events were hyponatremia (4%), infection (4%) and sensory neuropathy (4%).

**Supplemental Table SI.** Patient demographics and baseline clinical characteristics by DTI

| Characteristic | Short DTI (n = 135) | | | Long DTI (n = 201) | | |
| --- | --- | --- | --- | --- | --- | --- |
|  | R-CHOP  (n = 97)  n (%) | EPOCH  (n = 38)  n (%) | *P** | R-CHOP  (n = 150)  n (%) | EPOCH  (n = 51)  n (%) | *P*** |
| Age (years)  Median (range)  > 60 years | 73 (34 - 89)  83 (86) | 65 (23 - 75)  22 (58) | < 0.01 | 71 (39 - 92)  117 (78) | 65 (29 - 80)  35 (69) | 0.19 |
| Sex  Male | 61 (63) | 20 (53) | 0.33 | 75 (50) | 25 (49) | 1.00 |
| Stage  III-IV | 88 (91) | 35 (92) | 1.00 | 92 (61) | 33 (65) | 0.74 |
| ECOG PS  > 1 | 48 (50) | 18 (47) | 0.85 | 21 (14) | 16 (31) | 0.01 |
| Extranodal site(s)  > 1 | 49 (51) | 25 (66) | 0.13 | 46 (31) | 21 (41) | 0.17 |
| Serum LDH  Elevated | 83 (86) | 34 (90) | 0.78 | 84 (56) | 39 (77) | 0.01 |
| B symptoms  Present  Unknown | 43 (44)  0 | 23 (62)  1 | 0.08 | 21 (14)  2 | 10 (20)  1 | 0.43 |
| Size  ≥ 10 cm | 11 (12) | 5 (13) | 0.77 | 7 (5) | 4 (8) | 0.48 |
| COO (Hans)  GCB  Non-GCB  Unknown | 25 (32)  54 (68)  18 | 5 (17)  24 (83)  9 | 0.16 | 48 (40)  73 (60)  29 | 13 (33)  27 (68)  11 | 0.46 |
| IPI  Low  Low-int  High-int  High | 8 (8)  8 (8)  20 (21)  61 (63) | 3 (8)  4 (11)  11 (29)  20 (53) |  | 36 (24)  39 (26)  48 (32)  27 (18) | 11 (22)  12 (24)  8 (16)  20 (39) |  |

DTI, diagnosis-to-treatment interval; R-CHOP, rituximab, cyclophosphamide, doxorubicin, vincristine, and prednisolone; EPOCH, dose-adjusted etoposide, prednisolone, vincristine, cyclophosphamide, doxorubicin and rituximab; ECOG PS, Eastern Cooperative Group Performance Status; LDH, lactate dehydrogenase; COO, cell-of-origin; GCB, germinal center B; IPI, International Prognostic Index; int, intermediate.

*R-CHOP vs. EPOCH in the short DTI group, **R-CHOP vs. EPOCH in the long DTI group.

**Supplemental Table SII.** Grade 3/4/5 adverse events of the patients in the long DTI group by treatment regimen

| Adverse events | R-CHOP (n = 150) | | | DA-EPOCH-R (n = 51) | |
| --- | --- | --- | --- | --- | --- |
|  | Grade 3  n (%) | Grade 4  n (%) | Grade 5  n (%) | Grade 3  n (%) | Grade 4  n (%) |
| Hematologic |  |  |  |  |  |
| Neutropenia | 21 (14) | 66 (44) | 0 | 2 (4) | 39 (76) |
| Leukopenia | 35 (23) | 54 (36) | 0 | 3 (6) | 35 (69) |
| Thrombocytopenia | 12 (8) | 7 (5) | 0 | 12 (24) | 10 (20) |
| Anemia | 15 (10) | 2 (1) | 0 | 22 (43) | 2 (4) |
| Febrile neutropenia | 16 (11) | 2 (1) | 0 | 18 (35) | 1 (2) |
| Nonhematologic |  |  |  |  |  |
| AST increased | 2 (1) | 1 (1) | 0 | 1 (2) | 0 |
| ALT increased | 2 (1) | 1 (1) | 0 | 1 (2) | 0 |
| Hyponatremia | 1 (1) | 1 (1) | 0 | 1 (2) | 1 (2) |
| Constipation | 0 | 0 | 0 | 1 (2) | 0 |
| Nausea | 1 (1) | 0 | 0 | 1 (2) | 0 |
| Infection | 5 (3) | 3 (2) | 0 | 2 (4) | 0 |
| Allergic reaction | 0 | 0 | 0 | 0 | 0 |
| Tumor lysis syndrome | 0 | 0 | 0 | 0 | 0 |
| Sensory neuropathy | 2 (1) | 0 | 0 | 2 (4) | 0 |
| Motor neuropathy | 0 | 0 | 0 | 1 (2) | 0 |
| Pneumonitis | 3 (2) | 0 | 1 (1) | 1 (2) | 0 |
| Thrombosis | 0 | 0 | 0 | 0 | 1 (2) |
| Others | 0 | 1 (1) | 0 | 3 (6) | 0 |

DTI, diagnosis-to-treatment interval; R-CHOP, rituximab, cyclophosphamide, doxorubicin, vincristine, and prednisolone; DA-EPOCH-R, dose-adjusted etoposide, prednisolone, vincristine, cyclophosphamide, doxorubicin and rituximab; AST, aspartate aminotransferase; ALT, alanine aminotransferase.

Others: In patients treated with the R-CHOP regimen, Grade 4 creatine phosphokinase increased (n = 1) was were observed. In patients treated with the DA-EPOCH-R regimen, grade Grade 3 gastrointestinal disorders—Other, specify (bowel movement) (n = 1), Grade 3 hemorrhoids (n = 1), and Grade 3 mucositis (n = 1) were observed.
