# Supplementary material for: Impact of the diagnosis-to-treatment interval on the survival of patients with CD5-positive diffuse large B-cell lymphoma
Source: Ann Hematol. 2026 Apr 25;105(5):267. doi: 10.1007/s00277-026-07021-0 (PMC13110205; doi:10.1007/s00277-026-07021-0)
Supplement: Supplementary file 2 — Supplementary file2 (PPTX 165 kb) Figure SI. Kaplan‒Meier curves of the progression-free survival and overall survival of patients stratified by DTI grouped by week for the entire cohort (a, b), for the patients who received R-CHOP (c, d), and for the patients who received DA-EPOCH-R (e, f). DTI, diagnosis-to-treatment interval; R-CHOP, rituximab, cyclophosphamide, doxorubicin, vincristine, and prednisolone; DA-EPOCH-R, dose-adjusted etoposide, prednisolone, vincristine, cyclophosphamide, doxorubicin, and rituximab [file 277_2026_7021_MOESM2_ESM.pptx]

## Slide 1
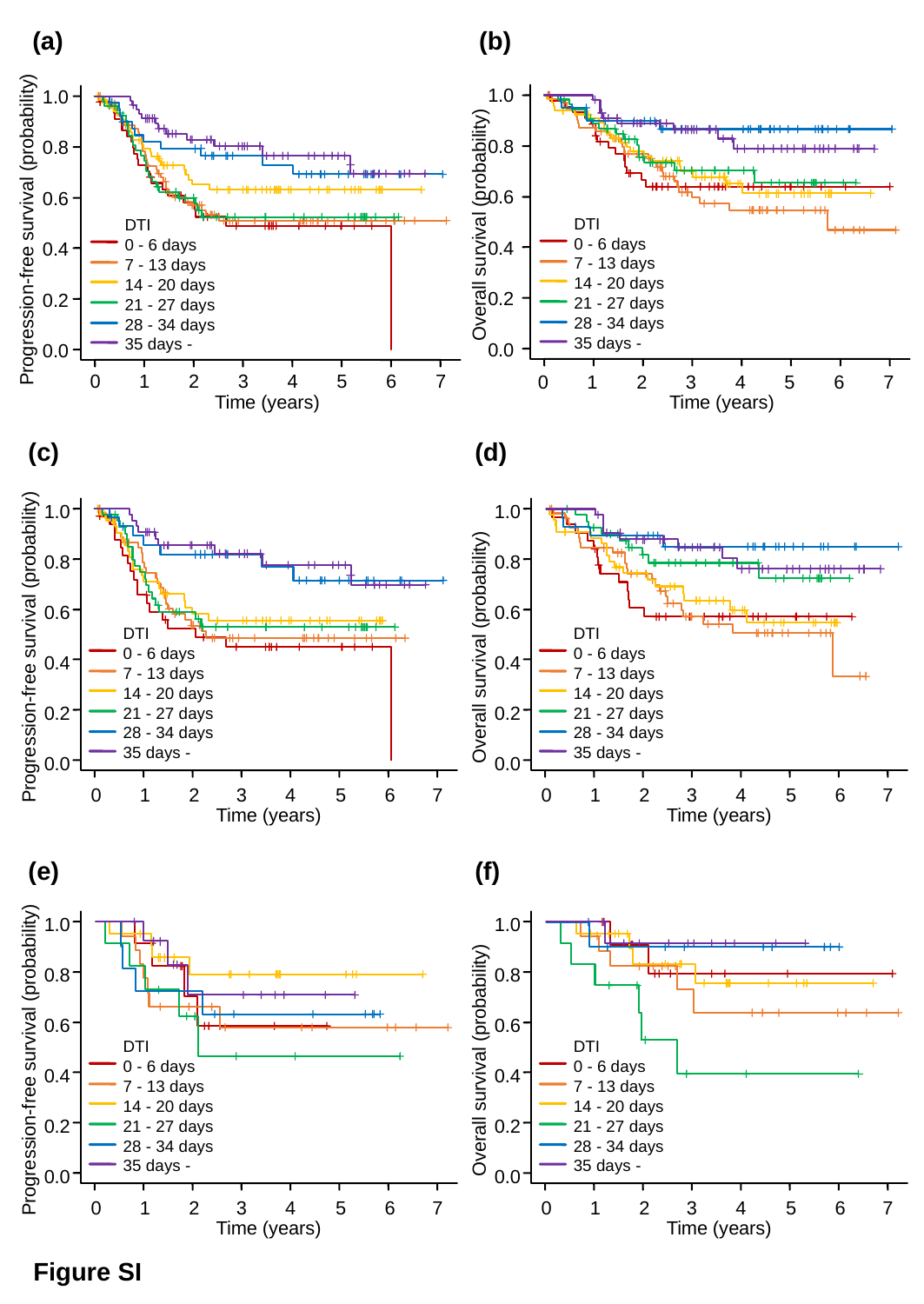

(a)
(b)
1.0
0.8
0.6
DTI
0 - 6 days
7 - 13 days
14 - 20 days
21 - 27 days
28 - 34 days
35 days -
Progression-free survival (probability)
0.4
0.2
0.0
0
1
2
3
4
5
6
7
Time (years)
1.0
0.8
0.6
DTI
0 - 6 days
7 - 13 days
14 - 20 days
21 - 27 days
28 - 34 days
35 days -
Overall survival (probability)
0.4
0.2
0.0
0
1
2
3
4
5
6
7
Time (years)
(c)
(d)
1.0
0.8
0.6
DTI
0 - 6 days
7 - 13 days
14 - 20 days
21 - 27 days
28 - 34 days
35 days -
Progression-free survival (probability)
0.4
0.2
0.0
0
1
2
3
4
5
6
7
Time (years)
1.0
0.8
0.6
DTI
0 - 6 days
7 - 13 days
14 - 20 days
21 - 27 days
28 - 34 days
35 days -
Overall survival (probability)
0.4
0.2
0.0
0
1
2
3
4
5
6
7
Time (years)
(e)
(f)
1.0
0.8
0.6
DTI
0 - 6 days
7 - 13 days
14 - 20 days
21 - 27 days
28 - 34 days
35 days -
Progression-free survival (probability)
0.4
0.2
0.0
0
1
2
3
4
5
6
7
Time (years)
1.0
0.8
0.6
DTI
0 - 6 days
7 - 13 days
14 - 20 days
21 - 27 days
28 - 34 days
35 days -
Overall survival (probability)
0.4
0.2
0.0
0
1
2
3
4
5
6
7
Time (years)
Figure SI
