# Supplementary figures and images for: Impact of the diagnosis-to-treatment interval on the survival of patients with CD5-positive diffuse large B-cell lymphoma
Source: Ann Hematol. 2026 Apr 25;105(5):267. doi: 10.1007/s00277-026-07021-0 (PMC13110205; doi:10.1007/s00277-026-07021-0)

## Slide 1
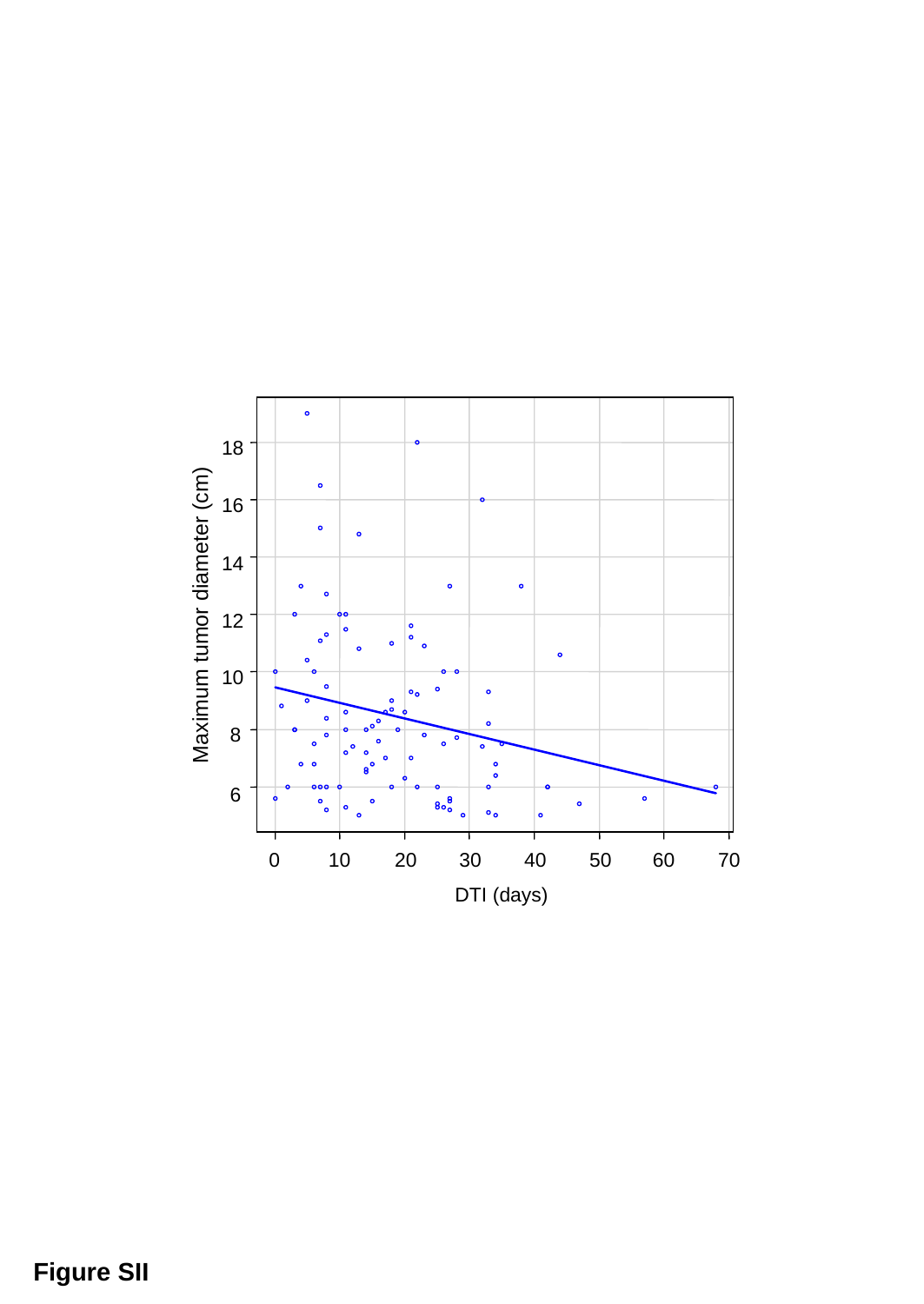

18
16
14
12
10
8
6
0
10
20
30
40
50
60
70
Maximum tumor diameter (cm)
DTI (days)
Figure SII

Supplement: Supplementary file 3 — Supplementary file3 (PPTX 27 kb) Figure SII. Correlations between the DTI and the maximum tumor diameter were determined using Spearman’s rank correlation analysis. DTI, diagnosis-to-treatment interval [file 277_2026_7021_MOESM3_ESM.pptx]
