# Supplementary material for: Impact of the diagnosis-to-treatment interval on the survival of patients with CD5-positive diffuse large B-cell lymphoma
Source: Ann Hematol. 2026 Apr 25;105(5):267. doi: 10.1007/s00277-026-07021-0 (PMC13110205; doi:10.1007/s00277-026-07021-0)
Supplement: Supplementary file 4 — Supplementary file4 (PPTX 106 kb) Figure SIII. Distributions of the DTI of patients who received R-CHOP (a) and DA-EPOCH-R (b). Kaplan‒Meier curves of the progression-free survival and overall survival of patients who received R-CHOP compared with those who received DA-EPOCH-R in the short DTI group (c, d) and in the long DTI group (e, f). DTI, diagnosis-to-treatment interval; R-CHOP, rituximab, cyclophosphamide, doxorubicin, vincristine, and prednisolone; DA-EPOCH-R, dose-adjusted etoposide, prednisolone, vincristine, cyclophosphamide, doxorubicin, and rituximab [file 277_2026_7021_MOESM4_ESM.pptx]

## Slide 1
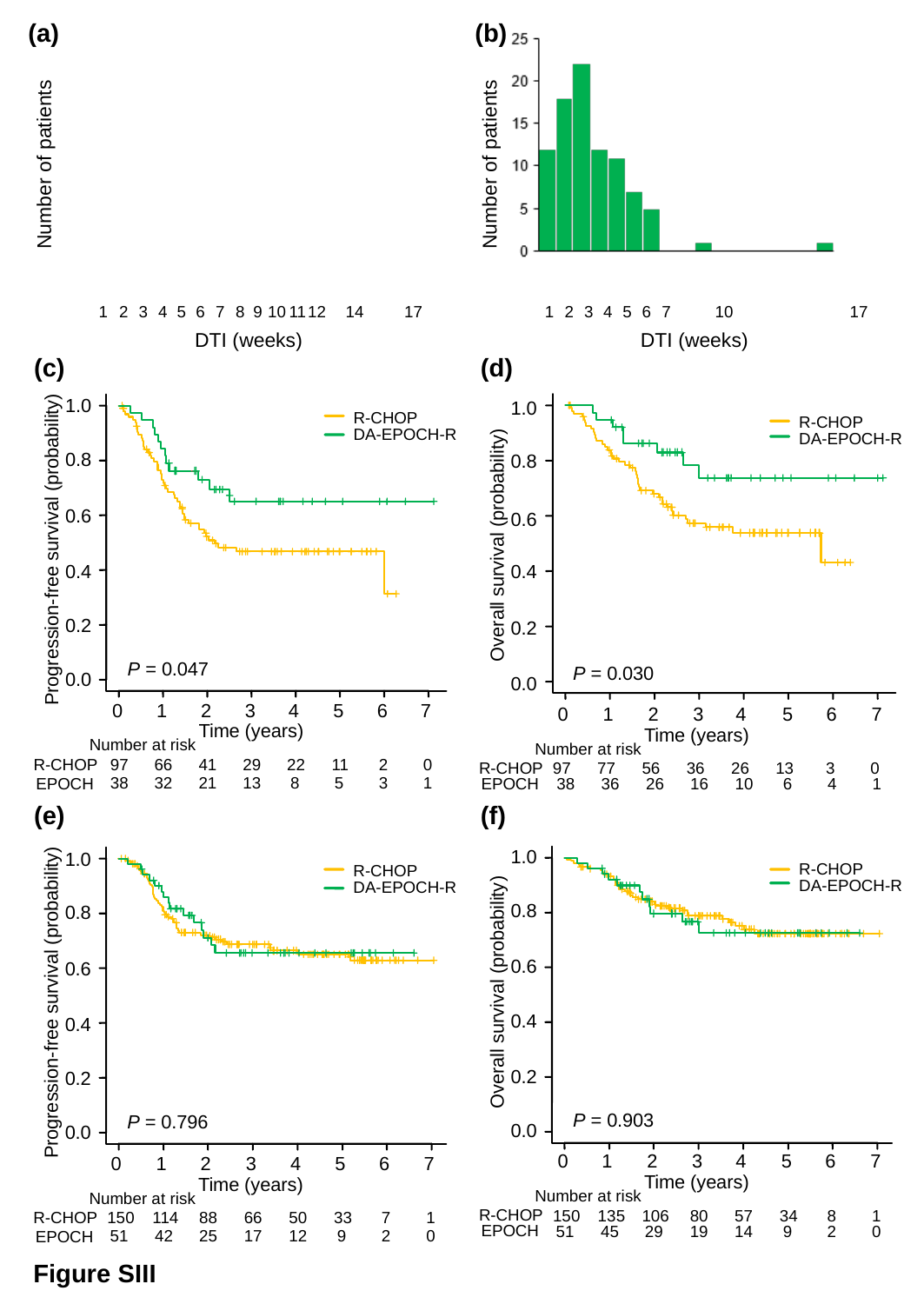

(a)
(b)
Number of patients
1
2
3
4
5
6
7
8
9
10
11
12
14
17
DTI (weeks)
Number of patients
1
2
3
4
5
6
7
10
17
DTI (weeks)
(c)
(d)
1.0
1.0
R-CHOP
DA-EPOCH-R
0.8
0.6
0.4
0.2
P = 0.030
0.0
0
1
2
3
4
5
6
7
Time (years)
Number at risk
R-CHOP
97
77
56
36
26
13
3
0
EPOCH
38
36
26
16
10
6
4
1
Overall survival (probability)
R-CHOP
DA-EPOCH-R
0.8
0.6
Progression-free survival (probability)
0.4
0.2
P = 0.047
0.0
0
1
2
3
4
5
6
7
Time (years)
Number at risk
R-CHOP
97
66
41
29
22
11
2
0
38
32
21
13
8
5
3
1
EPOCH
(e)
(f)
1.0
R-CHOP
DA-EPOCH-R
0.8
0.6
Progression-free survival (probability)
0.4
0.2
P = 0.796
0.0
0
1
2
3
4
5
6
7
Time (years)
Number at risk
R-CHOP
150
114
88
66
50
33
7
1
51
42
25
17
12
9
2
0
EPOCH
1.0
R-CHOP
DA-EPOCH-R
0.8
0.6
Overall survival (probability)
0.4
0.2
P = 0.903
0.0
0
1
2
3
4
5
6
7
Time (years)
Number at risk
R-CHOP
150
135
106
80
57
34
8
1
EPOCH
51
45
29
19
14
9
2
0
Figure SIII
